# Emotional states predict cellular immune system activity under conditions of life as it is lived: A multivariate time-series analysis approach

**DOI:** 10.1371/journal.pone.0290032

**Published:** 2023-11-09

**Authors:** Lennart Seizer, Dietmar Fuchs, Harald R. Bliem, Christian Schubert

**Affiliations:** 1 Department of Child and Adolescent Psychiatry, Psychosomatics and Psychotherapy, University Hospital of Tübingen, Tübingen, Germany; 2 Institute of Psychology, University of Innsbruck, Innsbruck, Austria; 3 Department of Psychiatry, Psychotherapy, Psychosomatics and Medical Psychology, Medical University Innsbruck, Innsbruck, Austria; 4 Division of Medical Biochemistry, Biocenter, Medical University Innsbruck, Innsbruck, Austria; University of Roehampton - Whitelands College, UNITED KINGDOM

## Abstract

The relationship between emotional states and immune system activity is characterized by bidirectional influences; however, limited information is available regarding the temporal dynamics of these effects. The goal of this investigation was to examine how these psychoimmunological interdependencies unfold over time under conditions of “life as it is lived”. For this purpose, three healthy women collected their entire urine over a period of approximately two months at 12-h intervals (8 am–8 pm, 8 pm–8 am), resulting in a total of 112 to 126 consecutive measurements per subject. In addition, among other regular psychological assessments, the subjects completed the EWL-60-S, an emotional state questionnaire, each morning and evening. To assess the extent of T-helper type 1 immune activation, the neopterin per creatinine concentration was measured in the urine samples using high-pressure liquid chromatography. The dynamic relationships between the time series of the six emotional states (performance-related activity, general inactivity, extraversion/introversion, general feeling of comfort, emotional irritation, anxiety/depressiveness) and urinary neopterin levels were estimated in vector-autoregressive models and evaluated using Granger-causality tests, impulse-response functions and forecast error variance decompositions. The findings showed that emotional states explained up to 20% of the variance of urinary neopterin per creatinine levels, whereby most of the effects occurred within a period of approximately three days. Across all subjects, increases in anxiety/depressiveness and extraversion led to increases in neopterin levels, while a general feeling of comfort led to decreases in neopterin. These results emphasize the importance of the interdependencies between emotional states and immune system activity and showcase the potential that intensive longitudinal study designs offer for psychoneuroimmunology.

## 1. Introduction

Psychoneuroimmunology (PNI) is an interdisciplinary field exploring the complex interactions between psychological processes and immune system activity [1]. PNI has challenged the predominant paradigm of autonomous functioning of the endocrine, nervous, and immune systems advocating an integrative biopsychosocial approach [2]. Conventional immunology describes several phenomena autonomously. However, complete understanding of the immune system in a living organism requires a broader theoretical perspective considering that various components of the organism are interacting to maintain a dynamic state of homeostasis while being functionally challenged by internal and external stimuli [3]. In the following, we argue that the dynamic interactions within this network can hardly be investigated by a cross-sectional approach relying on the aggregation of single measurements from different subjects; instead, they require a dynamic perspective that enables us to investigate temporally lagged relations and to handle within- and between-subject variation adequately [4].

A major research topic in PNI has been the relationship between emotional states and immune system activity. Emotions are multidimensional phenomena comprising cognitive, behavioral, expressive, experiential, and physiological components that operate in a coordinated and synchronized manner. They are generated and maintained in a dynamic transactional process between organisms and internal or external stimuli and have evolved to increase the adaptability of human responses to environmental challenges [5, 6]. In this context, multiple influences of emotional states on immune system activity have been described and are mediated by patterns of neurological activity that trigger downstream pathways with various endocrine effectors, for instance, the hypothalamic‒pituitary‒adrenal (HPA) axis and the sympathetic nervous system (SNS) [7–9]. Among these influences, the best described are likely the proinflammatory responses to acute fear and stress, emotions that have evolutionarily evolved as reactions to situations that pose a threat of an injury to the body. Thus, the upregulation of immune activity along with these psychological states can be advantageous to combat possible injuries or infections resulting from dangerous situations in a timely and effective manner [10]. Positive emotions, on the other hand, might reduce levels of systemic inflammation either directly [11, 12] or by acting as a buffer against the adverse effects of negative emotional states (stress–buffer hypothesis) [13, 14]. Conversely, certain cytokines (e.g., IL-1, IL-6, TNF-α) can also pass the blood‒brain barrier (BBB) and influence cognitive and emotional aspects of human experience and behavior [11, 15].

Most prior studies into the relationship between emotions and immune system activity have been conducted either in a laboratory setting using an emotion-eliciting protocol or by using recall questionnaires, in which subjects retrospectively report on their emotions over the past weeks or months. Thus, information about nuanced relationships between emotions and the immune system, especially in terms of the temporal dynamics of effects during everyday life, remains limited. However, the timing of measurements to determine the effect of emotions on immune activity is not trivial: Graham-Engeland et al. [16] found stronger trends of association between momentary negative affect reports and inflammatory cytokines when negative affect was assessed closer in time to cytokine measurement. Furthermore, some previous studies have applied an ambulatory repeated-measurement design and have shown that the duration of these psychophysiological effects can last for several days, thus surpassing the temporal scope of many laboratory studies. Schubert et al. [17, 18] found that emotionally meaningful positive and negative everyday incidents are followed by cyclic responses in urinary cortisol and neopterin levels that last for several days; Maydych et al. [19] reported a brief naturalistic stressor to influence monocyte counts and functionality one week afterward; and Schubert and Hagen [11] identified bidirectional relationships between IL-6 levels and emotional states covering intervals between 12 h and 72 h.

Attempting to capture the dynamic interplay between emotions and immune activity through aggregated snapshot-like assessments in a laboratory setting or by connecting retrospective timeline-recall measures of emotions with momentary inflammation levels presents significant challenges. The application of such single-time or short-term measurement designs implicitly assumes (i) a temporal stationarity of the variables examined, (ii) a relative time-invariance of the system under study (i.e., study results from time t_1_ are applicable at a different time t_2_) and (iii) a between-subject homogeneity in temporal stimulus-response delays (i.e., study results from person p_1_ are applicable to a different person p_2_) [20]. However, these assumptions are not applicable for the relationship between emotions and the immune system. First, emotional and immunological activity exhibit nonstationary features such as inherent rhythms of multiple lengths, including circadian, circaseptan, monthly, and yearly patterns [21–24], which can lead to spurious results if not accounted for [25]. Second, because emotional and immunological states are highly dependent on situational influences of ever-changing environmental demands (e.g., stress, injury) [26], the adaptational (emotional and immunological) responses to such incidents can change depending on the timing of the incidence and the current state of the individual. For example, due to diurnal changes in stress-system reactivity, the timing of stressor exposure can affect the elicited stress responses [27], and the time of day of vaccine injection influences the magnitude of the antibody response [28]. Third, the delay in onset and the duration of these emotional and immunological responses to certain challenges have been shown to vary between subjects [7, 17, 18, 29, 30], which makes it necessary to apply a repeated-measurement design to detect patterns within and between subjects.

Following the above considerations, the current investigation utilizes temporal data sets from a series of three integrative single-case studies performed under conditions of “life as it is lived” [18]. In these studies, three healthy subjects completed amongst other psychological assessments an emotional state questionnaire twice daily (each morning and evening) over a period of approximately two months. In addition, subjects collected their entire urine in 12-h intervals for the measurement of neopterin per creatinine levels. Neopterin is released by human monocyte-derived macrophages and dendritic cells in large amounts upon stimulation by interferon gamma (IFN-γ), a proinflammatory cytokine reflecting the T-helper type 1 (Th1) immune activation status [31]. Its urinary levels mirror the general biosynthesis of neopterin in the body since it is not biologically active, does not bind to receptors and undergoes rapid renal clearance [32].

The time series (i.e., repeated equidistant measurements) of emotional states and urinary neopterin concentrations were then used to examine psychoimmunological interdependencies via multivariate time-series analysis methods. Specifically, idiographic vector autoregressive (VAR) modeling was applied. The statistical analyses were performed intraindividually for each subject separately, i.e., based on the measurements of one subject at different points in time, and afterward, the results were compared interindividually [33]. Furthermore, in using VAR, a temporal order can be established between the variables that allows the inference of effective directionality and the investigation of how effects trace out over time [34–36]. Collecting data from subjects in the everyday lives, with the aim of minimizing interference with their daily routines and natural course of life, provides unparalleled ecological validity and helps to avoid common challenges associated with extrapolating findings from laboratory to field [37].

## 2. Methods

### 2.1 Study design

Using the integrative single-case design [18, 38] in a multiple case study framework [33], three healthy women were asked to collect time-series data to investigate psychoimmunological dynamics under everyday life conditions (“life as it is lived”). The application of time-series analytical methods often requires equidistant measurements, meaning that the data points are evenly spaced in time. This raises the important question of how to determine the sampling frequency in any time-series study. In this respect, factors such as the variability of variables and the expected delay of cause‒effect relations should be considered. In this study, measurements were taken in 12-h day-night intervals for reasons of feasibility (trade-off between study duration and sampling frequency) and because emotional states and immune system parameters show diurnal variation.

Following thorough initial psychological evaluation, the subjects collected their entire urine for two months in 112–126 consecutive 12-h intervals (equivalent of 56–63 days) from approximately 8 am to 8 pm (day) and from approximately 8 pm to 8 am (night) in polyethylene canisters. Upon collection, Na-Metabisulfite and Na-EDTA were added to each canister to prevent urine sedimentation and oxidation. Immediately after collection, the urine was aliquoted by each subject into polyethylene tubes, which were stored at -20°C at home. Once a week, these samples were brought to the laboratory where they were stored at -80°C until further analysis (maintaining an uninterrupted cold chain). Urine has several advantages as an analytical tool in repeated-measurement designs due to its abundant availability in large quantities and the noninvasively self-collection by the subjects [39]. Furthermore, at the end of each 12-h collection period (at approximately 8 am and 8 pm), subjects completed a diary-like set of questionnaires (Daily Inventory of Activity, Routine and Illness ☯DIARI]; [38]) in which they retrospectively reported on daily activities and routines (e.g., emotional states, caffeine and alcohol intake, physical activity, menstrual cycle) and signs of illness over the past 12 h. Also, subjects participated in weekly, in-depth interviews to assess emotionally meaningful positive and negative daily incidents from the previous week. This comprehensive approach provided equidistant (12-hour) time-series data. The present investigation focused on the time series of emotional states and urinary neopterin concentrations of the three subjects.

All subjects provided written informed consent for their participation and for the publication of data. The Ethics Committee of the University of Innsbruck approved this study.

### 2.2 Subject descriptions

Three healthy women were included as subjects in this study. All were living in Tyrol, Austria, were students and were of a similar age. Subject 1, a 25-years-old, participated in the study for 63 days (126 12-h intervals); Subject 2, a 27-years-old, participated in the study for 63 days (126 12-h intervals); and Subject 3, a 27-years-old, participated in the study for 56 days (112 12-h intervals). The three subjects had to meet the following inclusion criteria: good health, age after puberty and before menopause, native German speaker, and residence in or near Innsbruck, Austria. Pregnancy was an exclusion criterion. All of them were nonsmokers and did not use oral contraceptives. None of the subjects had any signs of medical or psychiatric symptoms at study entry, as determined by thorough physical examination, psychiatric evaluation and the *Check-up für Normalpersonen*, a questionnaire containing 33 items covering all major fields of medicine, including complaints that may be related to medical or psychiatric disease [40].

### 2.3 Emotional states

Each morning and evening, i.e., at the end of a 12-h unit, emotional states were assessed by each of the three subjects using the *Eigenschaftswörterliste* (EWL-60-S), a German paper-and-pencil questionnaire [41]. The EWL-60-S captures emotional states in six categories (*performance-related activity*, *general inactivity*, *extraversion/introversion*, *general feeling of comfort*, *emotional irritation*, *anxiety/depressiveness*) with a total of sixty items/adjectives (see Table 1). Each item was rated on a four-point Likert scale (1 = *not at all*, 2 = *a little*, 3 = *quite*, 4 = *very*). The summed scores for each category were calculated by adding up the ratings of each category’s items and dividing this sum by the number of items in the category. Thus, each category’s score ranges between 1 and 4. The category *extraversion*, consisting of a total of eight items, includes four items that measure introversion, and these scores are inverted prior to summation. The EWL 60-S is recommended for use in longitudinal designs. Its Cronbach’s alpha is between 0.4 and 0.86 [41]. The test requires approximately 5 min to complete and is given before any other psychological measures and before urine aliquotation to ensure that external factors do not influence emotional state reporting.

**Table 1:** EWL-60-S emotional state categories. The mean and standard deviation (SD) of the six emotional state categories of the *Eigenschaftswörterliste* (EWL-60-S) are given for each of the three subjects. The last column contains the items of each category, translated from German. *These item scores are inverted.

	Mean ± SD			
Category	S1	S2	S3	Items
Performance related activity	2.35 ±0.36	2.15 ±0.5	1.90 ±0.38	*forceful*, *alert*, *active*, *concentrated*, *stable*, *eager*, *persevering*, *energetic*
General inactivity	1.21 ±0.31	1.55 ±0.39	1.31 ±0.39	*without energy*, *foggy-brained*, *limp*, *dozy*, *worn down*, *sleepy*, *sluggish*, *dazed*, *weak*, *tired*, *drowsy*, *weary*
Extraversion/Introversion	2.92 ±0.33	2.38 ±0.63	2.97 ±0.42	shy*, *trusting*, taciturn*, *talkative*, isolated*, *outgoing*, monosyllabic*, *sociable*
General feeling of comfort	2.39 ±0.43	2.17 ±0.59	2.19 ±0.53	*jolly*, *self-assured*, *self-content*, *cheerful*, *easygoing*, *joyous*, *glad*, *self-confident*
Emotional irritation	1.20 ±0.31	1.11 ±0.2	1.14 ±0.26	*irritated*, *agitated*, *sensitive*, *angry*, *irritable*, *annoyed*, *fidgety*, *nervous*, *vulnerable*, *furious*, *upset*, *fragile*
Anxiety/Depressiveness	1.08 ±0.17	1.05 ±0.14	1.19 ±0.35	*anxious*, *saddened*, *miserable*, *uneasy*, *sad*, *frightened*, *fearful*, *worried*

### 2.4 Biochemical analysis

In the urine samples of each of the three subjects, neopterin levels were measured using high-pressure liquid chromatography (HPLC; Model LC 550; Varian Associates, Palo Alto, CA, United States), normalized against urinary creatinine (HPLC) and expressed as μmol neopterin per mol creatinine to account for variations in urine density [42]. All urine aliquots of each of the three subjects were measured in one single run within three months following collection. A new aliquot was used for each of at least three independent determinations.

### 2.5 Time-series analysis

All analyses were conducted in *R* 4.2 using the packages *tseries* and *vars* [43–45]. The dynamic relationship between the time series of emotional states and urinary neopterin concentrations was investigated using a multivariate technique called vector autoregression (VAR) [46]. A VAR model consists of a set of regression equations for multiple (two or more) time-series variables, including time-lagged values. All variables are treated as endogenous, which means that each variable is considered both a predictor and the outcome of the other variables in the system. Nevertheless, as influences that are determined outside the system of interest might also affect the variables in the VAR, other variables that are considered to have a purely exogenous effect can be included as well. In the following, an explanation of the procedures of VAR model estimation and interpretation is given [34, 46, 47].

Prior to calculating a VAR, each included time series in the model should be tested for *stationarity*. In time-series analysis, stationarity refers to a variable’s constant mean and variance over time, which can be tested using the augmented Dickey-Fuller (ADF) test along with visual inspection of the corresponding time-series plot and autocorrelation function (ACF). The first important step in VAR modeling is *lag-order selection*, which refers to the number of lagged values considered in the model. The optimal lag order is determined by using model selection criteria, such as the Akaike Information Criterion (AIC) [34]. After estimating a VAR with a certain lag order, several *diagnostic tests* should be performed to confirm that the model meets all assumptions [34]. Among these are the Jarque-Bera test for the normal distribution of the residuals and the autoregressive conditional heteroscedasticity (ARCH) test for variance stationarity. To verify that the residuals were free of autocorrelation, the Portmanteau test was conducted and the ACFs were visually inspected. The stability of the model was tested according to the eigenvalue stability condition.

In contrast to usual regression analyses, the raw coefficients are difficult to interpret in a VAR, as the variables’ dynamics are a result of the behavior of the system at once. Thus, certain methods are used to analyze and interpret the relationships and influences of the variables within the system: Granger-causality tests, impulse-response functions, and forecast error variance decomposition. *Granger-causality* can be used to describe the cause‒effect relation between two variables X and Y over time. If, after accounting for past values of Y, the inclusion of values from variable X improves the prediction of future values from variable Y, it is said that variable X Granger-causes variable Y [48]. This technique is generally believed to improve causal inference compared to analyses in cross-sectional data [49]. *Impulse response functions* (IRF) are based on the VAR model and depict the time-lagged response of one variable to an isolated, simulated increase in another variable while controlling for the influence of all other variables in the VAR. Confidence intervals (given as bootstrapped error bands) of 68% have been recommended for IRF [50, 51]. To ensure that only the most conservative IRF estimations are reported, the order of the variables was adjusted before the computation of each IRF, whereby the respective variable was entered as the rightmost column of the data frame to account for the effects of all other variables prior to the one of interest [47]. *Forecast error variance decomposition* (FEVD) estimates the amount of variance in one variable that can be explained by the impact of other variables in the system at a certain forecast horizon (temporal lag). In this work, we analyzed the amount of variance in urinary neopterin levels that can be explained by the emotional states at different lags (up to 10 lags).

## 3. Results

### 3.1 Descriptive statistics

**Fig 1:**
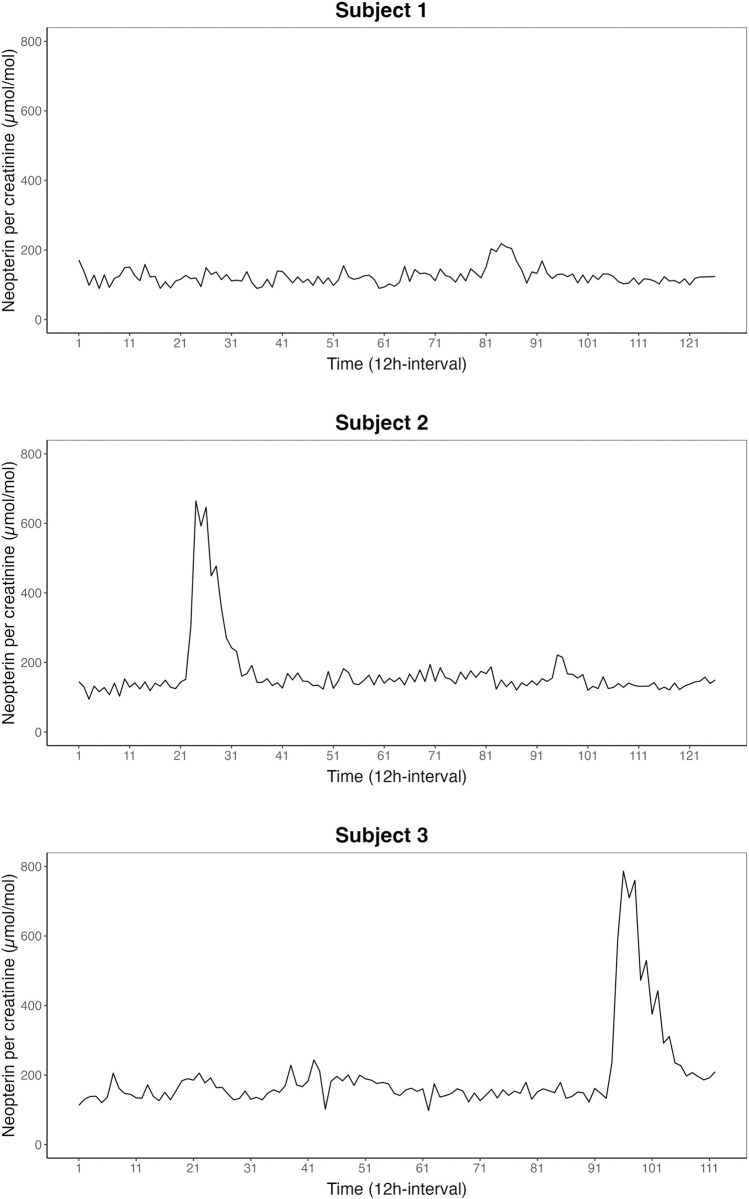
Time series of urinary neopterin per creatinine levels. The levels of urinary neopterin per creatinine in μmol per mol over the course of the study period are depicted for each of the three subjects. The three time series covered periods of 112 to 126 12-h time units during which the subjects collected their entire urine in day portions (8 am–8 pm, uneven numbers) and night portions (8 pm–8 am, even numbers).

The time-series of urinary neopterin per creatinine concentrations from the consecutive 12 h-pooled urine samples are depicted in Fig 1. For Subject 1, neopterin concentrations averaged *M* = 123.63 (*SD* = 24.05) μmol/mol; for Subject 2, *M* = 167.79 (*SD* = 89.99) μmol/mol; and for Subject 3, *M* = 193.90 (*SD* = 120.07) μmol/mol. The urinary neopterin per creatinine nighttime levels were significantly higher than the daytime levels for Subject 1 (*t* = -3.61, *p* < 0.01) and Subject 2 (*t* = -2.99, *p* < 0.01), but no diurnal difference was found for Subject 3 (*t* = 1.24, *p* = 0.22). Descriptive statistics on the six emotional state categories are given in Table 1.

### 3.2 Granger causality

The Granger causality between the variables under study was tested in bivariate analyses using a lag order of 2 to match the underlying diurnal frequency of the data. In Subject 1, the prediction of neopterin levels significantly improved by including values of extraversion (*F* = 3.15, *p* = 0.04). The urinary neopterin levels of Subject 2 were significantly Granger-caused by anxiety/depressiveness (*F* = 5.94, *p* < 0.01), extraversion (*F* = 9.96, *p* < 0.01), performance-related activity (*F* = 6.02, *p* < 0.01) and general feeling of comfort (*F* = 3.31, *p* = 0.04), while Subject 3 did not show significant results in the bivariate analyses.

### 3.3 VAR model estimation

The variables included in the VAR models were urinary neopterin per creatinine concentrations (Fig 1), the six emotional state categories (Table 1), and the following exogenous variables to control for third-variable influences: alcohol and caffeine intake, physical activity, menstrual cycle and a binary dummy variable for diurnal variation. For each subject, a separate VAR model was fitted. Both Subject 2 and Subject 3 fell ill during the study period, and they experienced symptoms associated with stomach flu. Since the reason for this aberration in neopterin levels was exogenous to the system under study, a dichotomous variable was constructed that indicated either the presence (‘1’) or absence (‘0’) of outliers in each neopterin time series to control for this influence in the VAR. Outliers were defined as those that were greater than two interquartile ranges from the central 50% of the data [52]. These control variables were included in the VAR models as an additional exogenous impact. The optimal VAR lag-order was *p* = 1 for all subjects. The resulting VAR models passed all diagnostic tests.

### 3.4 Impulse response functions

**Fig 2:**
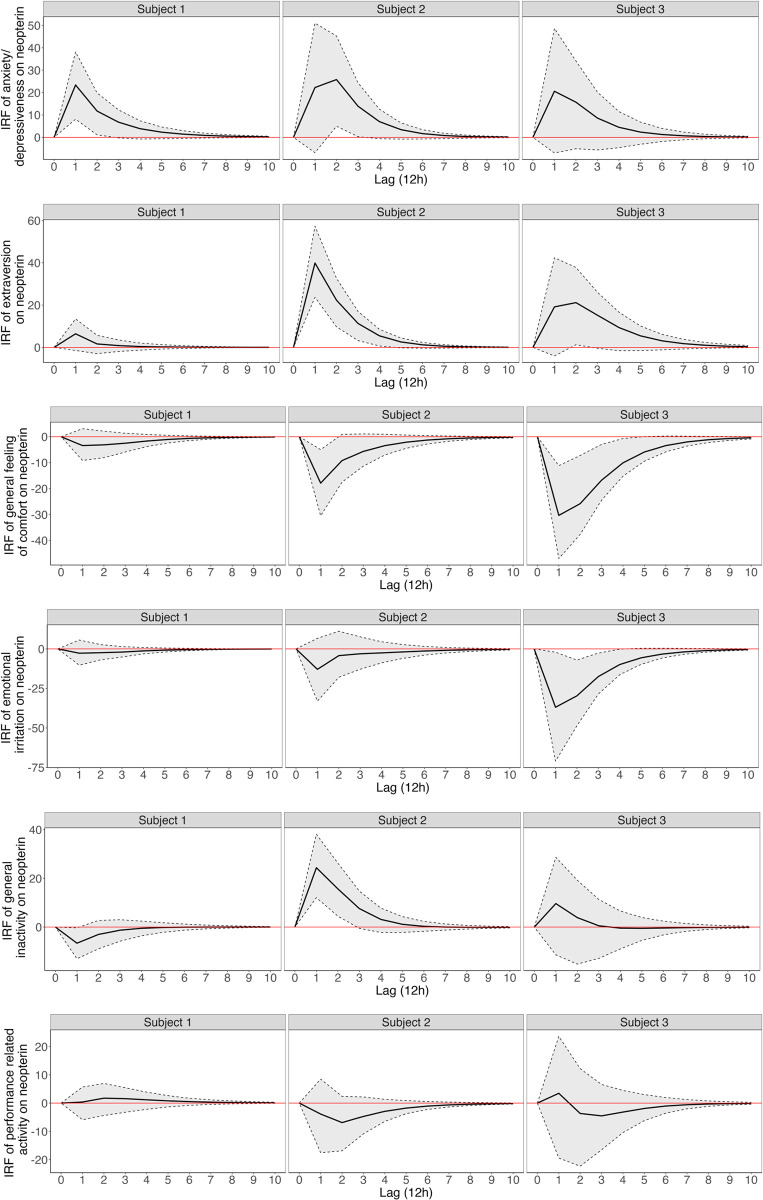
Impulse response functions. For each of the three subjects (columns), the impulse-response function (IRF) of the 6 emotional state categories on urinary neopterin per creatinine levels (rows) is given for 10 lags. One lag represents a 12-h interval. In each function, a shock is simulated in the respective emotion category with neopterin as the response variable. A shock represents an increase of one standard deviation in the impulse variable. The response is considered significant when the error bands do not include zero.

Fig 2 shows the IRFs for each subject, which trace out the dynamic response of urinary neopterin concentrations over ten lags (five days) to an isolated shock in each of the six emotional states categories. A shock represents an increase of one standard deviation in the impulse variable. The response is considered significant when the error bands do not include zero. Generally, the subjects had similar response trajectories in neopterin for impulses in most emotional state categories but showed differences in the effect sizes, significance and duration of the response. Specifically, impulses in anxiety/depressiveness and extraversion led to an increase in urinary neopterin concentrations in all subjects. These associations showed variable durations, with significant neopterin increases occurring up to 24–36 h (2 lags) later in Subject 1 and Subject 2 for anxiety/depressiveness and significant neopterin increases up to 36–48 h (3 lags) later in Subject 2 and Subject 3 for extraversion. On the other hand, urinary neopterin levels tended to decrease after impulses in the general feeling of comfort, with significant neopterin decreases in both Subject 2 and Subject 3 up to 36–48 h (3 lags) later. Performance-related activity did not show any significant temporal pattern. The effects of general inactivity and emotional irritation on neopterin were more inconsistent across subjects.

### 3.5 Forecast error variance decomposition

**Fig 3:**
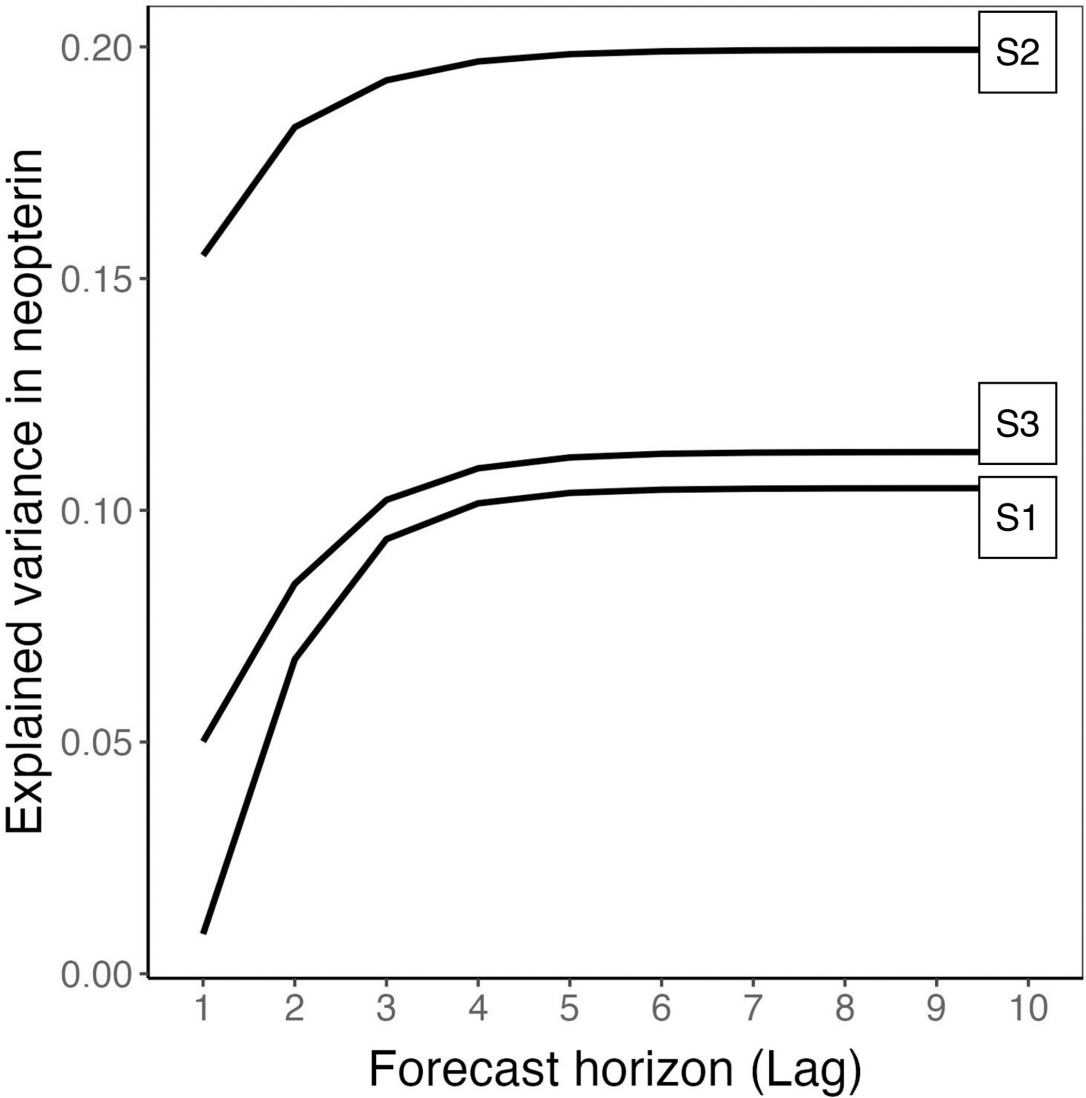
Forecast error variance decomposition. Each line depicts the amount of variance explained in urinary neopterin per creatinine levels by emotional states for each of the three subjects at various forecast horizons/temporal lags.

The results of the FEVD for urinary neopterin concentrations at different lags are given in Fig 3. In all subjects, the explained variance of neopterin by the emotional states grows with an increasing forecast horizon until approximately lag 6, i.e., 72–84 h. After this temporal horizon of approximately three days, the explained variance becomes relatively stable for all subjects, which means that most effects of emotions on neopterin are exerted within this period. At this horizon, the emotional states of Subject 1, Subject 2, and Subject 3 explain 19.90%, 10.44% and 11.22% of the variance in urinary neopterin levels, respectively.

## 4. Discussion

In this series of integrative single-case studies [18], three healthy women collected their entire urine in pooled 12-h interval portions for neopterin per creatinine measurement and, among other regular psychological assessments, completed a questionnaire on emotional states twice a day over a period of approximately two months. The influence of emotional states on urinary neopterin concentrations was estimated using VAR models. Across all subjects, elevated levels of anxiety/depressiveness and extraversion led to increases in the neopterin per creatinine concentration over the following days, while increases in performance-related activity and general feelings of comfort led to decreases in the neopterin per creatinine concentration (Fig 2). In the FEVD, we found that the emotional states accounted for up to approximately 20% of the variance in neopterin, with most of the effects being exerted within 6 lags, i.e., approximately three days (Fig 3). These long-lasting effects might be explained by the fact that emotional states experienced during everyday life often relate to personally meaningful themes (e.g., relationship, work) and ebb and flow without clear temporal boundaries compared to emotional responses observed in experiments, which are often standardized and time-limited.

The result that an increase in anxiety/depressiveness predicts increases in Th1 immune activation is in line with previous studies that assessed negative emotional states and immune activity [53, 54]. Similarly, depression and anxiety disorders have also been associated with increased levels of peripheral inflammation markers in patients, although the immune activity in healthy subjects and in patients with affective disorders should be compared with caution [55]. We also found increased extraversion to be followed by increased levels of Th1 immune activation across all subjects. This association has been shown previously for trait levels and might be explained by the fact that extraversion is associated with outgoing behavior and social interactions, which in turn pose an increased exposure to antigens and elevated risk of injury [56, 57]. Performance-related activity and general feelings of comfort levels tended to be followed by decreased Th1 immune activation. These categories might be associated with states of confidence and self-efficacy (see items in Table 1), which have been shown to positively influence immune system functioning [58].

Intensive longitudinal data and time-series analysis allow us to model the relationships of psychoimmunological variables over time and to establish a temporal order in the way that current changes in one variable lead to future changes in another variable. Therefore, questions on directionality that result from correlational research might be disentangled [47]. Furthermore, effects that occur with a temporal lag can be detected and compared across individuals. However, importantly, even a significant relationship over time does not imply causality between two variables, as there might be a third variable driving the relationship that has not been accounted for in the study. Furthermore, the VAR approach presented in this work enables the detection of short-lagged effects but is insensitive to relationships that unfold over long-range timescales (e.g., trends), which would require vector error correction models [34]. Nevertheless, time-series approaches provide value in the clarification of relationships and mechanisms of psychological and biological variables that dynamically interact in the emergence of multidimensional psychobiological phenomena such as emotions.

While for most of the emotional states, the general direction of effect on urinary neopterin levels was consistent across the three subjects, there were considerable differences in the dynamic structure of the effect, such as the delay to onset, the peak and the duration (Fig 2). These variations likely emerged through (i) differences in the emotion-evoking incidents that each subject encountered during the study period and (ii) between-subject differences in trait emotionality, personality, and coping styles. For example, the modalities of a stressor such as its quality, magnitude, duration, timing, and novelty, can alter neuroendocrine responses subsequently influencing the immune system [27]. Furthermore, personality types and traits in emotion regulation have been shown to not only determine stress system reactivity and functioning but also modulate the effect of emotions on the immune system [57, 59]. Hence, in further multiple case studies it may be beneficial to incorporate qualitative analyses of everyday incidents and subject characteristics. Another limitation of the present study is the use of only neopterin as a surrogate marker of the Th1 immune response. In future studies, additional components of the stress and immune system, such as cortisol, IL-6, and tumor necrosis alpha (TNF-α), might be measured, as they may react differently to emotional states and can provide deeper insights into the molecular mechanisms leading to stress-related neopterin changes.

In a nomothetic research approach, data are usually generated and aggregated across individuals from samples as large as possible with the inherent assumption of between-subject homogeneity. This approach has the advantage of potential generalization in the respective population (if certain criteria for size and representativeness of the sample and replication of the results are met). However, due to the aggregation, it may only identify the most common characteristic or mechanism within the sample, diluted by heterogeneous subjects. A faulty conclusion is that these average characteristics can then be inferred for all subjects in the sample (termed *ecological inference fallacy*) [20]. Thus, effects found in large-scale studies often have low implicative meaning for individuals, and furthermore, effects that are small or absent at a population level might be of importance in specific individuals [20, 30]. For instance, 27% of patients with depression show elevated inflammatory mediators [60] which does not preclude the possibility that, for some patients, depressive symptoms might be fully induced by or completely unrelated to inflammation. Especially in the study of psychoimmunological processes, interindividual heterogeneity is more typical than exceptional even in “homogeneous” samples and likely contains valuable information about the processes under study. Thus, as Richard Lazarus stated, “we need to understand human variation if we are to deal effectively with individuals” [5, p. 53].

## Supporting information

S1 Data (CSV)
